# Violence Prevention Climate and Health-Oriented Leadership in German Emergency Departments

**DOI:** 10.3390/healthcare11162234

**Published:** 2023-08-08

**Authors:** Mannat Guliani, Sonja Reißmann, Joachim Westenhöfer, Volker Harth, Stefanie Mache

**Affiliations:** 1 Department of Health Sciences, Hamburg University of Applied Sciences (HAW), Ulmenliet 20, 21033 Hamburg, Germany; mannat.guliani@haw-hamburg.de (M.G.); joachim.westenhoefer@haw-hamburg.de (J.W.); 2 Institute for Occupational and Maritime Medicine (ZfAM), University Medical Center Hamburg-Eppendorf (UKE), Seewartenstraße 10, Haus 1, 20459 Hamburg, Germany; s.reissmann@uke.de (S.R.); harth@uke.de (V.H.)

**Keywords:** workplace violence, leadership, emergency department, health-oriented leadership, violence prevention climate

## Abstract

Emergency departments (EDs) are high-risk environments for workplace violence. Research into techniques to prevent violence has less frequently explored the influence of leadership. This study aims to analyze the association of leadership with the prevention of violence using the concepts of health-oriented leadership (HoL) and the violence prevention climate (VPC). This quantitative cross-sectional study was conducted through online surveys between November 2021 and March 2022 across Germany. A sample of 370 doctors and nurses working in German EDs were recruited. Perceptions towards VPC and HoL were compared between groups divided according to profession and position using independent *t*-tests or Mann–Whitney *U* tests. Separate multiple linear regression models for supervisors and employees analyzed the association between different profiles of HoL with VPC. Supervisors and employees showed significant differences in supervisor staff-care and VPC. Regression analysis demonstrated that supervisors’ self-care and employees’ assessment of supervisor’s staff-care positively predicted all dimensions of VPC. This empirical study provides insights into the variable perceptions of different groups and the association of leadership profiles with the perceptivity of VPC. The results of this study can be used to emphasize the importance of HoL training for both employees and supervisors to improve communication and health-promoting behavior.

## 1. Introduction

Workplace violence or occupational violence can be defined as “any action, incident or behavior that departs from reasonable conduct in which a person is assaulted, threatened, harmed, injured in the course of, or as a direct result of, his or her work” [1] (p. 4). The global prevalence of any type of workplace violence against healthcare workers (HCWs) in emergency departments (EDs) is 79.4% (95% Confidence Interval [CI] 75.2–83.6) as per a recent systematic review [2]. ED employees across the world experience the most events of non-physical violence (62.3%, 95% CI 53.7–70.8). Physical violence is most prevalent in mental health settings (50.6%, 95% CI 34.8–66.4) and second most common in EDs (31%, 95% CI 26.0–36.0) [2]. Studies in Germany estimate the frequency of violence in EDs as 97.1% of employees experiencing verbal abuse and 87.4% physical abuse from patients. On the other hand, 64.5% are exposed to physical violence from relatives and 94.3% verbal aggression from relatives [3].

Despite many prevention strategies, HCWs, especially those working in the EDs, often accept workplace violence as a part of their job, something that is unavoidable [4,5,6]. The perception of inevitability and powerlessness is partly because HCWs do not perceive active involvement of the organization or leadership in the prevention of violence [5,6]. Also, only very few studies [4,7] solely focus on investigating the role of leadership in the prevention of workplace violence according to the authors’ best knowledge.

There is no defined or accepted benchmark to measure and describe a good leader in the ED as the leaders here require a range of attitudes [8]. In the field of health and clinical medicine, probably only EDs present employees and leaders with the need to take decisions, each second of the day, all day, all year. This is a myriad of chances that can lead to success or failure. Employees in the ED work in these intimidating working conditions with a usual lack of time and functioning methodology. This structure of the EDs necessitates the need for efficient ED leaders who empower their teams to acquire coping strategies to function despite these limitations [8]. However, it is critical to note that research about leadership in EDs is in its inception stage [9,10].

On the other hand, a significant role of leadership in creating climate is well documented [11], essentially proposing that “leaders create climate” [12] (p. 1519), [13]. The relationship between leadership and safety climate is found in many studies [12,14,15,16]. There is a suggested need for supervisors to become “champions of workplace safety” [17] (p. 83). Leaders who practice passive leadership might assume that non-action regarding safety issues is harmless, but it can lead to negative consequences. A comparison of passive and safety-specific transformational leadership with safety climate demonstrates the negative impact of the former [17]. Although these studies provide evidence of the essentiality of leadership, there is still a need to explore its association with climate, especially concerning violence prevention. This exploration becomes even more consequential in EDs because workplace violence is highly prevalent [2,3], and leadership is under-researched [9,10]. Therefore, the present study uses two concepts, health-oriented leadership, and violence prevention climate, with an aim to analyze their association.

### 1.1. Violence Prevention Climate

The concept of a perceived violence climate or violence prevention climate is developed from the idea of safety climate [18]. VPC is the perception of employees about the measures enforced by the organization against occupational violence [18]. A positive perception, i.e., a positive VPC could mean that the management focusses on reducing the frequency of violence and increasing the potential for identifying risk factors at an early stage, thereby preventing violence better [18,19]. It is made up of three dimensions: practices and response, policies and procedures, and pressure for unsafe practices [19]. The first construct, ‘practices’ entails the employee’s evaluation of the management’s compliance with the official violence prevention policies. It also includes assessing their ‘response’ to such episodes. The second construct, ‘policies’, is designed to examine employee’s awareness of the operational violence-prevention policies in the organization. This dimension also comprises ‘procedures’ used by the organization to communicate this information to its employees as it is equally important. Both these constructs together are essential for a good violence prevention climate. A lack of awareness about relevant policies would not be useful and advertising strong policies but disregarding them when faced with violent events could reinforce aggression. On the other hand, employees may not be aware of strong policies but may witness practices demonstrating expected behavior. Therefore, both policies and practices would positively influence the VPC. The third construct, ‘pressure for unsafe practices’ identifies neglect or disregard of violence prevention policies in order to adhere to the group if violence is not considered abnormal by peers, or to comply with other conditions. For instance, an important determinant negatively affecting a good violence prevention climate could be a strong emphasis on completing the tasks from the supervisor without caring for prevention. In such a case, the violence prevention climate would be perceived negatively by the employee, even with the existence of effective policies [19].

As per the authors’ best knowledge, existing studies on VPC are yet to look for differences in these perspectives among different populations on account of their position, or profession. On the flip side, previous safety climate studies have analyzed and found differences, for instance, between clinical leaders and frontline clinicians with respect to patient safety and the teamwork climate [20]. Therefore, with an objective to address this research gap, this study hypothesizes the following:

**H1.**  *The ratings for all three dimensions of VPC (practices and responses, policies and procedure, pressure for unsafe practices) differ significantly between respondents based on their position in the ED (supervisor/employee) and their profession (doctor/nurse).*

### 1.2. Health-Oriented Leadership

The HoL model was developed to explain health-specific leadership behavior in a more comprehensive way than the general models of leadership [21]. The HoL model can be used to study the impact of leaders and the role of followers in healthy leadership. Health-oriented leadership differentiates between the leadership of self (self-care/self-directed health-oriented leadership) and staff (staff-care/follower-directed health-oriented leadership), based on the perspective of both supervisors and employees [21]. The model is divided into five constructs for external and self-assessment: the assessment of self-care by both the employee and supervisor, staff-care by the supervisor, the employee assessment of staff-care of a supervisor (perceived staff-care) and the employee perception of supervisor self-care [22]. Each of these constructs further has three dimensions of value, awareness, and behavior. Value is the worth associated with health or how important health is to leaders or employees; awareness refers to the attention given to health risks, and the factors that influence health; behavior can be described as the rules or actions implemented for the improvement in health [21,22]. The behavior component is further analyzed through the perspective of motivation in personal lifestyle, and the promotion of healthy behavior at work [23].

Previous studies report that perceived supervisor staff-care is associated with lesser burnout, depression, musculoskeletal problems, and increased self-care, thereby promoting health in employees [24]. This staff-care perceived by employees is also negatively related to burnout and alleviates the negative impact of work efforts on burnout [25]. There may be an association between staff well-being and perceived staff-care in terms of more job satisfaction, commitment, engagement, and decreased strain [26]. A recent study concluded that employee ratings of their supervisor’s staff-care in terms of awareness, value and behavior can significantly predict their depression and anxiety levels, while the supervisor’s personal assessment of their staff-care is not significantly associated with the average mental condition of the team [27].

The health-oriented leadership framework can be utilized to compare different professional groups [28]. Moreover, because the ratings can be obtained from both supervisors and supervisees, “reciprocal dyadic leader-follower relationships” can be investigated [29] (p. 10). Such types of comparisons can help accentuate the strengths and weaknesses of each group based on the difference or similarity of their perception to improve leadership [28]. There may be differences in how leaders and employees perceive the staff-care that they give and receive, respectively [27].

The second objective of this study is to add evidence to the existing body of research on comparisons between different groups with regard to HoL, specifically in EDs. Therefore, this study hypothesizes the following:

**H2.**  *The ratings for staff-care and self-care differ significantly based on the position of respondents in the ED (supervisor/employee) and based on their profession (doctor/nurse), there are significant differences in self-care, supervisor staff-care and the employee assessment of supervisor staff-care.*

### 1.3. Health-Oriented Leadership and Violence Prevention Climate

Many aspects of health-oriented leadership such as behavior of the leader, for instance, in response to an employee being sick would be observable by others and would act as a signal to their team according to the signaling theory [30,31]. Employees must interpret signals and behavior patterns based on the selected characteristics of policies, practices, and pressure to perceive climate [12]. These signals help employees perceive what kind of behavior is “expected, rewarded and supported” [12] (p. 1518). Additionally, according to the social learning theory, when employees notice their supervisors’ awareness or behavior towards health, they assess the organizational environment, reinforcing what is acceptable [32,33].

If both concepts of VPC and HoL are analyzed together, it can be presumed that a leader’s health-oriented leadership behavior would send signals that may aid employees in the interpretation of a violence prevention climate. Here, the research question evolves on whether the profiles of health-oriented leadership shape perceptions of the violence prevention climate. With the objective to answer this question, this study analyzes the association of different profiles of health-oriented leadership with the violence prevention climate.

Based on previous research, just as leaders’ transformational leadership is known to be associated with the safety climate [16,17], this study assumes that employees’ assessment of their supervisor staff-care would be associated with their perceived violence prevention climate. This is in addition to their own perspectives towards healthcare having an effect. This means that how they take care of their own health and how they perceive to be taken care of might predict how they perceive the violence prevention climate of their organization. Additionally, the model of supervisors assumes that how supervisors take care of themselves and how they think they take care of their staff would be associated with how they perceive the violence prevention climate of their organization. Therefore, this study hypothesizes:

**H3.**  *Supervisor ratings for the self-care and staff-care variables are associated with violence prevention practices, violence prevention policies, and pressure for unsafe practices.*

**H4.**  *Employee ratings on the variables of self-care and assessments of supervisor staff-care are associated with violence prevention practices, violence prevention policies, and pressure for unsafe practices.*

## 2. Materials and Methods

### 2.1. Study Design and Recruitment of Participants

A quantitative study with a cross-sectional design was used for the purpose of this research. Primary data were collected through an online survey in Germany from November 2021 to March 2022.

Four criteria were defined to aid in the inclusion and exclusion of the participants. The first inclusion criterion was the presence of an independent and spatially separate emergency department in the hospital. Second, a minimum of three months’ work experience at the current facility. The first two criteria were included on the assumption that time less than three months or temporarily working in EDs might not give the participant enough perspective. Additionally, participants had to be working either as a doctor or a nurse to be eligible for this study. This criterion was included to exclude employees who either solely performed administrative tasks or worked in emergency rescue services. The last was the pre-condition of working in direct contact with patients or their attendants to assess their experiences with external workplace violence caused by patients and their attendants.

Participants were recruited in two stages one after the other. In the first stage, a provisional list provided by the Federal Joint Committee (Gemeinsamer Bundesausschuss, G-BA) assigning 1751 German EDs to their respective care level [34] was used. The G-BA has established a graduated system to classify German EDs into four care levels: 0 (minimal or specialized care centers), 1 (basic care), 2 (advanced care) and 3 (comprehensive care) [35]. To achieve an adequate and representative sample, a double-probability sampling method that included both systematic and stratified sampling (see Table S1) was used to create recruitments lists, shortlisting 211 hospitals (G-BA 1 = 125, G-BA 2 = 53 and G-BA 3 = 33). A larger category (G-BA 0), with a total of 683 hospitals, was excluded from recruitment as EDs in this category may not have a separate emergency ward. Information regarding the email addresses of the Chief Medical Officers (German: Chefarzt:in) and the nursing leaders (German: pflegerische Leitung) of these emergency departments was acquired either via the hospital websites or telephone. However, 48 hospitals had to be excluded from recruitment either because of (i) unwillingness to participate in any study, (ii) faulty email addresses, (iii) absence of a central emergency department in the hospital or (iv) failure to establish a contact to acquire the required email addresses. Therefore, recruitment was extended and eventually, a total of 210 EDs were contacted between 22 November to 14 December 2021, and with 124 G-BA level 1, 53 G-BA level 2 and 33 G-BA level 3 hospitals. Reminders were sent between 10 and 25 January 2022. By 10 February, 248 answers had been received.

In the second stage, contact with the central directory of German emergency departments [36] was established. This directory was established by the working group for the registry and care research from the department of traumatology at the University Hospital Magdeburg on behalf of the German Interdisciplinary Association for Intensive and Emergency Care (DIVI e.V.) and the German Society for Interdisciplinary Emergency and Acute Medicine (DGINA e.V.). With help of the directory management, emails with the survey details were sent to 850 EDs on February 11, making it a convenience sample.

Data were collected via LamaPoll, a German online survey platform. Multiple responses were controlled by enabling cookies in the survey settings. The survey was online and open for participation from 17 November 2021 to 08 March 2022. At the end of the survey period, out of 878 visitors on the survey link, 540 started the questionnaire and 391 completed it.

### 2.2. Variables and Measurements

#### 2.2.1. Sociodemographic Variables

Nine self-constructed questions for demographics were included in the questionnaire. Items for age and lengths of experience had a single-choice format while shift work was a multiple-choice question. Gender, G-BA level, the category of funding for the hospital, and location of the ED in terms of federal state were single-answer questions. Leadership was a binary yes/no question. The number of beds in the participant’s hospital to estimate its size, was enquired by formulating a question based on a hospital report from Germany [37]. Another question was derived from the Copenhagen Psychosocial Questionnaire (COPSOQ)-16 to assess the number of working hours per week.

#### 2.2.2. Violence Prevention Climate

The Violence Prevention Climate Scale-18 or VPCS was developed as an 18-item scale [19]. The original English scale was a six-point Likert scale. For this study, a five-point scale (1 = Strongly disagree, 2 = Disagree, 3 = Neither agree nor disagree, 4 = Agree and 5 = Strongly Agree) was used instead. The scale was downloaded from the website [38] and translated to German by native German and English speakers. To adapt to the context of the present study, slight modifications were made for the German version after clarifying with Dr. Spector, i.e., “Reports of workplace violence from other employees are taken seriously by management” became “Reports of workplace violence from employees are taken seriously by management”, ‘cover-up for lawsuits’ was translated to ‘protection against lawsuits’, and the word ‘management’ was replaced by ‘clinic management’ (Klinikleitung).

The scale contained three dimensions that were separately analyzed, practices and responses, policies and procedures and pressure for unsafe practices, all of which had six items per sub-scale. The six items for the third dimension, ‘pressure’ had to be reverse coded so that high scores for any dimension of VPC always reflected a good violence prevention climate [19]. The original authors established the construct validity of this scale by using an exploratory factor analysis to finalize the current 3-factor structure of the scale. The criterion-related validity was substantiated by the association of less physical and psychological strain with a better climate [19]. The internal reliability or Cronbach’s α coefficients of the three dimensions for this study were calculated to be practices, α = 0.885, policies, α = 0.903 and pressure, α = 0.894 for the total sample. A sum score was calculated for each of the three VPC variables: practices, policies, and pressure as elaborated in the VPCS scoring instructions on the website for statistical analysis [38].

#### 2.2.3. Health-Oriented Leadership

The health-oriented leadership instrument is constituted of five scales. There are two for supervisors (i.e., self-care and staff-care) and three for employees (i.e., self-care, employee assessment of supervisor staff-care and employee assessment of supervisor self-care) [22]. The fifth scale, i.e., employee assessment of supervisor self-care, was not included in this study to reduce the length of the questionnaire as it was anticipated to be of least relevance to this study. The remaining four self-care and staff-care scales were composed of 19 and 22 items, respectively. The HoL instrument had to be answered on a five-point Likert scale, (1 = Not at all true, 2 = Slightly true, 3 = Moderately true, 4 = Very true, 5 = Completely true). Two items from all awareness sub-scales had to be reverse coded for all three scales [39]. All scales were reported to have adequate validity by the original authors. Content-related validity was verified through evaluation, construct validity through confirmatory factor analysis and tested with instruments of transformational leadership and abusive supervision, and criterion validity was verified through correlations with different health measures [22]. Reliability analysis in the present study was performed by calculating Cronbach’s alpha for all sub-scales and complete scales (see Table S2). One subscale having less than 2 items was computed using the Spearman–Brown coefficient, which is less biased in the case of a two-item scale [40]. All values ranged between 0.719 and 0.949. For analysis of HoL variables, three mean values were defined to summarize self-care, staff-care by supervisors and employee assessment of supervisor staff-care for each participant.

### 2.3. Statistical Methods

An a priori sample size of *n* = 175 was calculated using G*power software (version 3.1) for a multiple linear regression model with two predictors, effect size *f*^2^ = 0.09, alpha value α = 0.05 and power (1 − β) = 0.95. However, since separate models were created for supervisors and employees, the total sample size required was estimated to be *n* = 350.

Data analysis was conducted using IBM SPSS Statistics (version 27). Data from 14 participants were removed because they did not fulfil the inclusion criteria. Seven participants were excluded due to straightlining across one or more complete scales. Therefore, a final sample of *n* = 370 was considered for data analysis.

The study population was divided into categories either by position (supervisors and employees) or by profession (doctors and nurses) in accordance with hypotheses H1 and H2. Assumptions of normality, linearity and homogeneity were tested for all variables and groups. Outliers were removed, reducing the supervisor staff-care by two data points. Accordingly, parametric or non-parametric tests were conducted. An independent *t*-test with bootstrapping was used for comparing the ratings of supervisors and employees on violence prevention practices and self-care, as well as of doctors and nurses on supervisor staff-care. Additional analysis was performed using Mann–Whitney *U*-tests with a Monte-Carlo method based on 100,000 samples. As multiple comparisons between the same groups were carried out, the family-wise type I error was corrected using the Holm–Bonferroni procedure. For better estimates of effect size and uniformity throughout the study, Cohen’s *d* was reported for all tests.

For Hypothesis H3 and H4, six multiple linear regression models were created, three for supervisors and three for employees for each dimension of VPC. All assumptions for regression analysis were satisfied (see Table S3). Multiple test adjustments using the Holm–Bonferroni method were only performed for the ANOVA results of the six models as part of post hoc testing. Adjustments were not made for the regression coefficients in each model as they are not usually carried out for multiple regression analysis [41]. As both the dependent and predictor variables were continuous, an effect size, Cohen *f*^2^, was calculated [42] for each model. Post hoc testing with a Holm correction was used to compute an adjusted *p*-value for each model. An additional post hoc test with G*power software (version 3.1) was used to evaluate the models to calculate power.

## 3. Results

### 3.1. Descriptive Statistics

#### 3.1.1. Sociodemographic Data

A final sample of *n* = 370 participants working in EDs across Germany were part of this study. The sample was composed of 55.1% employees and 44.9% supervisors with their overall division into 112 doctors and 258 nurses. Most participants were female (59.5%), aged between 30 and 39 years (26.8%), working in a publicly funded hospital (57.0%) for 1–5 years (41.3%). Further details about participants and settings have been listed in Table 1.

#### 3.1.2. Health-Oriented Leadership and Violence Prevention Climate

**Figure 1:**
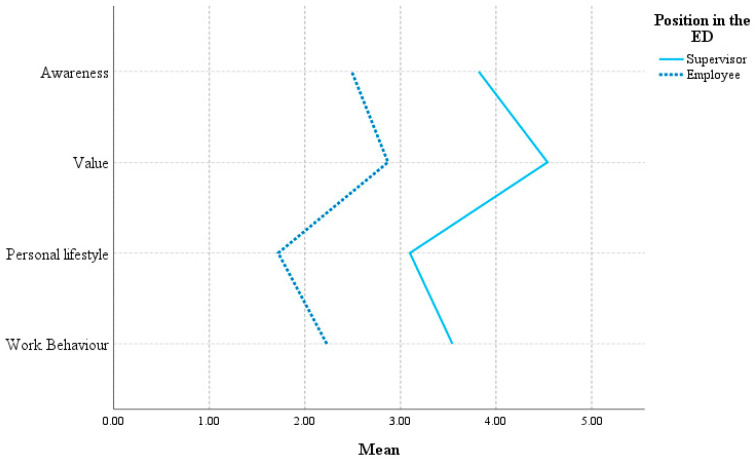
Mean ratings of supervisors and employees on staff-care.

**Figure 2:**
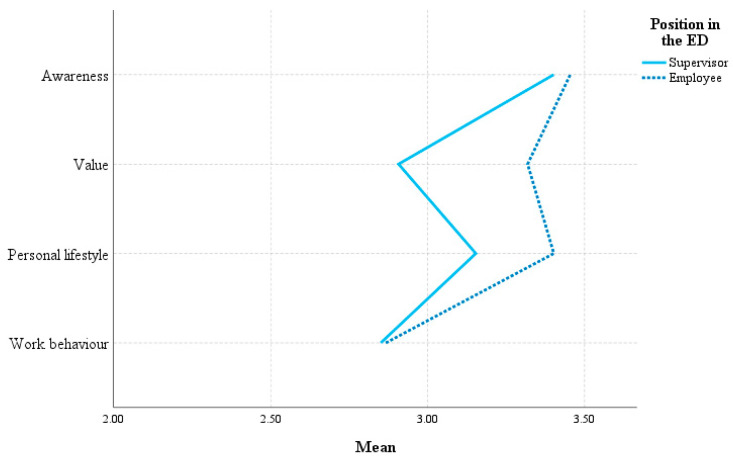
Mean ratings of supervisors and employees on self-care.

HoL variables were multi-dimensional, with the underlying dimensions of awareness, value, personal lifestyle, and work behavior (see Table S4 for descriptive statistics per sub-scale). These dimensions were not individually analyzed except comparing the mean values for supervisors and employees on the scales of staff-care (Figure 1) and self-care (Figure 2). Supervisors gave higher ratings to their staff-care along all four dimensions than the employees perceived it. Comparing supervisors and employees on self-care, employees appeared to give better ratings, especially to the value and personal lifestyle for their own health.

Six variables were used for both descriptive and inferential statistics (see Table S5 for descriptive statistics). These were the three main overarching HoL variables, self-care, supervisor staff-care and the employee assessment of supervisor staff-care and three variables of VPC, violence prevention practices and responses, policies and procedures and pressure for unsafe practices. Owing to the different scales of staff-care for supervisors and employees, i.e., supervisors’ perspective on their staff-care and employees’ assessment of supervisor staff-care, analysis was mostly separated for supervisors and employees. In the case of supervisors, from a range of six to 30, the values for VPC were found to have a minimum of six for policies, seven for practices and eight for pressure, with a maximum of 30 for all three. However, for employees, the minimum and maximum for all three variables of VPC was six and 30, respectively. The self-care variable for supervisors ranged from 1.74 to 4.89 and for employees from 1.63 to 4.79. Supervisor staff-care ranged from 2.27 to 4.86 and the employee assessment of supervisor staff-care had the lowest value of 1.00 up to 4.82. As seen in Table 2 and Table 3, all scales had good internal consistencies ranging from 0.860 to 0.888 for supervisors and from 0.850 to 0.949 for employees. For supervisors, all five variables were significantly correlated with each other with the highest correlation between violence prevention practices and policies (Spearman’s rho = 0.570, *p* < 0.01). For employees, a significant correlation of self-care was not seen with two variables, prevention policies and the employee assessment of supervisor staff-care. The highest correlation here was also between practices and policies (Spearman’s rho = 0.551, *p* < 0.01). Relationships between the five main variables were found to be linear at both levels of supervisors and employees.

### 3.2. Hypothesis Testing

#### 3.2.1. Group Comparisons

In line with Hypothesis 1 and 2, differences in perspectives for HoL and VPC variables according to different professions and positions in the ED were checked (see supplementary material Tables S6–S8 for descriptive statistics per group). An independent *t*-test with bootstrapping was used for comparing the ratings of supervisors and employees on violence prevention practices and self-care, and of doctors and nurses on supervisor staff-care (Table 4). On average, supervisors reported a significantly better rating for violence prevention practices than employees, *t* (368) = 3.99, 95% CI [2.811, 5.145] *p* = 0.001. A medium to large effect size was computed, *d* = 0.737.

The other comparisons were conducted with Mann–Whitney *U*-tests with a Monte-Carlo method (Table 5). The ratings for violence prevention policies (*U* = 11,752.0, *z* = −5.07, *p* < 0.001), pressure for unsafe practices (*U* = 11,190.50, *z* = −5.63, *p* < 0.001) and staff-care (*U* = 3458.00, *z* = −13.09, *p* < 0.001) also differed significantly between supervisors and employees with medium (−0.547), (−0.612) and large (−1.867) effect sizes, respectively. There was also a significant difference in ratings of doctors and nurses regarding violence prevention practices (*U* = 10,446.50, *z* = −4.24, *p* < 0.001), and pressure for unsafe practices (*U* = 10,311.00, *z* = −4.39, *p* < 0.001) with small–medium effect sizes. Post hoc calculations including Holm–Bonferroni-corrected *p*-values (see Tables S9 and S10 for details) and Cohen’s *d* have been given with each comparison in Table 4 and Table 5.

#### 3.2.2. Regression Analysis

Hypotheses 3 and 4 were tested using multiple regression models for supervisors and employees, respectively. The two ratings of the HoL scale (self-care and staff-care) were simultaneously entered as independent variables into three regression models each for supervisors (Table 6) and employees (Table 7) to predict violence prevention practices, policies, and pressure for unsafe practices. When looking at the estimates of model parameters, the direction of the regression coefficients for all predictors were positive in all models. The values of effect size Cohen *f*^2^, the adjusted *p*-value using Holm corrections (see Tables S11 and S12 for details) and the power for all six models are listed in Table 8.

##### Supervisors

Supervisor self-care and staff-care significantly predicted violence prevention practices, F(2, 161) = 10.01, *p* < 0.001 and adjusted *R*^2^ = 0.100 (Table 6, Model 1) with a small–medium effect size. Model 2 for violence prevention policies was also significant of the data, F(2, 161) = 11.65, *p* < 0.001 and adjusted *R*^2^ = 0.116, with both predictors being significant (Table 6, Model 2). A small–medium effect size was calculated for this model. The third model regarding pressure for unsafe practices was significant of the data, F(2, 161) = 7.54, *p* = 0.001 and adjusted *R*^2^ = 0.074 (Table 6, Model 3), with a small–medium effect size. The *t*-test associated with the unstandardized regression coefficient of staff-care was not significant (*p* = 0.211). However, the original model with both predictor variables was retained due to a reduction in the explanation of variance from 7.4% (adjusted *R*^2^) to 7.1% (*R*^2^, as a single predictor remained in the model on removal of the non-significant variable). Assumption testing for multicollinearity, autocorrelation, and distribution initially along with the use of bootstrapping made these results robust and the insignificant *t*-tests were considered to be non-erroneous.

Therefore, it was concluded that for supervisors, self-care and staff-care significantly predicted violence prevention practices and policies. Supervisor self-care was significantly predictive of pressure for unsafe practices. The highest variance in the case of supervisors was explained for violence prevention policies.

##### Employees

Employee ratings on the variables of self-care and assessment of staff-care were associated with pressure for unsafe practices as indicated through the results of Model 6, of which the data was significant, F(2, 201) = 20.35, *p* < 0.001 and adjusted *R*^2^ = 0.160. The *t*-tests associated with the regression coefficients for both variables were highly significant (Table 7, Model 6). The effect size was medium–large, *f*^2^ = 0.190, for this model and a post hoc power analysis using the parameters, with α = 0.017, and two predictors in G*power amounted to a power of > 0.999 (Table 8).

Model 4 for violence prevention practices was also significant of the data, F(2, 201) = 9.61, *p* < 0.001 and adjusted *R^2^* = 0.078 with a small–medium effect size. Self-care did not significantly (*p* = 0.055) predict practices. Removal of self-care from the model reduced the explained variance to 7.1% (*R*^2^). Therefore, as discussed before for Model 3, the self-care variable was not removed from the model (Table 7, Model 4). Model 5 for violence prevention policies was also significant of the data, F(2, 201) = 9.99, *p* < 0.001, with the adjusted *R*^2^ = 0.081. However, the *t*-test associated with the unstandardized regression coefficient of self-care was not significant (*p* = 0.187). Removal of this variable from the model increased the variance explained to 8.4% (*R*^2^). Therefore, due to the increase in the explanation of variance, the new model with a single predictor was retained in this study. Model 5 (Table 7) was, therefore, significant of the data, F(1, 202) = 18.46, *p* < 0.001 and *R*^2^ = 0.084, with a small–medium effect size.

Based on these results, for employees, self-care and the assessment of supervisor staff-care significantly explained variance in pressure for unsafe practices. Employee assessment of supervisor staff-care was also significantly associated with violence prevention policies and practices. The highest variance in the case of employees was explained for pressure for unsafe practices. Cross-validation of the regression models in Table S13.

## 4. Discussion

The aim of this study was to apprehend the association of leadership with the prevention of violence in emergency departments in Germany. In doing so, the concepts of health-oriented leadership and violence prevention climate were used. This study attempted to increase the research knowledge on these concepts by looking at the disparities in perceptions of violence prevention climates and leadership profiles among groups divided per their position and profession in the ED. Statistical analysis using independent *t*-tests or Mann–Whitney *U* tests followed by post hoc Holm–Bonferroni corrections led to confirmation of most of the hypotheses for VPC (H1) and the rejection of most for HoL (H2). This means that group differences were found in supervisors and employees regarding violence prevention practices, policies, pressure, and staff-care. Group differences were found in doctors and nurses only for practices and pressure. The main objective was achieved by using multiple linear regression models to analyze the association of HoL with VPC. Health-oriented leadership profiles of self-care and staff-care from supervisors and employees were used as independent variables to predict the three dimensions of violence prevention climate, resulting in six models. All models were significant of the data. For employees (H4), self-care and assessment of supervisor staff-care significantly explained the variance in pressure for unsafe practices. Employee assessment of supervisor staff-care was also significantly associated with policies and practices. In the case of supervisors (H3), self-care and staff-care significantly predicted practices and policies. Supervisor self-care was also significantly predictive of pressure for unsafe practices. The highest variance in the case of employees was explained for pressure for unsafe practices and for supervisors, regarding policies.

### 4.1. Group Differences for Violence Prevention Climate

Higher VPC ratings imply that the participants believe that the management prioritizes violence prevention over organizational restraints [43]. Furthermore, this perception then impacts their behavior for violence prevention, thereby catalyzing a reduction in violent incidents [43]. Supervisors rated all three dimensions of VPC—practices, policies, and pressure—higher than employees, indicating a better VPC perception. Even though supervisor and employee ratings do not necessarily refer to the same EDs, this suggests that employees are more likely to feel that the management is not creating or implementing policies effectively, and existing policies are ignored under pressure. This is one of the first studies to compare supervisor and employee perspectives on the violence prevention climate, and more research is needed. However, safety climate studies show similar discrepancies, with employees perceiving a more problematic safety climate than supervisors and senior managers in 92 US hospitals [44], more clinic leaders having a better safety climate perception than frontline clinicians in a study across seven European countries [20], and employees with managerial functions rating safety climate more positively than those without in a Swiss study [45].

Doctors rated German EDs significantly higher than nurses along the dimensions of practices and pressure. This may be attributable to nurses having more exposure to workplace violence, probably due to more direct contact [2,4,46,47,48]. However, previous research does not find violence exposure to be predictive of VPC [43]. A parallel can again be drawn with the existing safety climate research as VPC was developed on the basis of the safety climate [18,19]. Studies in 13 public hospitals in Northern China [49] and four public hospitals in Palestine [50] showed that nurses have better perceptions of the safety climate than doctors. However, studies in 10 Australian intensive care units [51], 33 Dutch EDs [52], 10 Swiss hospitals [45], and 2 German university hospitals [53] found that doctors have a more positive safety climate perception than nurses, which aligns with the present study’s results. Some studies speculate that doctors are more connected to management and, thus, perceive better support [53,54]. However, none of these arguments can be considered as compelling evidence to support the obtained results, and there is a need for more studies using VPC to generate substantial evidence. Nonetheless, the differences in perspectives between doctors and nurses highlight the need for targeted interventions to improve the violence prevention climate.

### 4.2. Group Differences for Health-Oriented Leadership

A large significant difference between the supervisors’ perception of their staff-care and the employees’ assessment of it was found, thereby only partially confirming H2 as all the other comparisons were non-significant. Although supervisors and employees do not necessarily rate the same EDs, these results confirm that managers perceive themselves to give more value to their staff’s health and be more aware while encouraging a better personal lifestyle and work behavior than the employees discern. These results are in line with a previous health-oriented leadership study performed across 11 different organizations in Germany, which proposed that these higher ratings could be a result of an overconfidence bias [27]. According to research, individuals do not usually evaluate themselves correctly, and they are even worse at evaluating others [55]. The overestimation theory can be used to explain supervisors giving higher ratings to themselves, i.e., in the case of harder tasks, people tend to overestimate their performance [55]. The difference could also be due to different risk perceptions by supervisors, such that they may consider health to be a personal responsibility or may lack awareness of risks [56]. This cultural or psychometric risk perception may influence managers’ belief in impacting staff health, leading to a discrepancy between the self- and external assessment of staff-care [23,56].

### 4.3. Health-Oriented Leadership as a Predictor of the Violence Prevention Climate

Regarding the association of health-orientation profiles of employees with the three dimensions of VPC, the results imply that when employees feel that they are being taken care of, i.e., their immediate supervisor is concerned about their health and well-being, they perceive the violence prevention climate of the organization in all three dimensions to be better. Due to the scarcity of research on these concepts together, comparisons were made with existing safety climate research and then analyzed with existing research on health-oriented leadership and violence. Many studies have found an association between leadership and safety climate perception [11,14,57,58], with leadership being a positive predictor of safety climate [15,16,17]. Research suggests that leadership is a climate antecedent, with supervisors’ concern for employee well-being translating to their actions that affect their perception of employees’ safety climate [12]. The current study’s results, therefore, align with these conclusions, indicating that high staff-care assessment by ED employees corresponds to a better violence prevention climate perception. Previous research indicates that perceived staff-care by employees is a crucial predictor of the climate, finding a significant relationship between perceived staff-care and team health climate, as health-oriented leadership can affect the entire team [30]. A leader’s health orientation may create a health climate within the team through employees’ interpretation of signals [31].

In the case of employees, the results also imply that employees’ concerns about their health and well-being can affect their perception of pressure for unsafe practices. Self-care may aid employees in taking decisions for their health, thereby empowering them to seek support in a timely manner to enhance their working conditions [30]. Therefore, an employee with high self-care perceives less pressure because they actively engage in transforming their stressors.

Looking at the health-orientation profiles of supervisors, the results provide an insight into the dearth of research on the association of supervisors’ health perspectives with their climate perceptions. This result agrees with a previous study that states, “the health-orientation of a leader is a promising construct worthy to be further explored” [59] (p. 9).

### 4.4. Strengths and Limitations

This is one of the first studies to examine associations of various health-oriented leadership perspectives with different dimensions of the violence prevention climate. It is also one of the first studies to examine both these variables in the context of German EDs. It adds to the research base by shifting from the exploration of the role of leadership in the safety climate to its role in violence prevention. This study also adds to the existing bodies of research on HoL and VPC individually by examining differences in group perspectives, i.e., doctors and nurses and those with or without leadership positions. Few previous studies on HoL have focused on organizational health climate and team health climate [30,60,61]. This study has a relatively high external validity because the sample size is adequate and covers EDs from all federal states of Germany. It includes perspectives of respondents from different age groups, genders, professions, working experiences, types of EDs, and geographical locations. However, this composition may not be representative in terms of some variables.

Even though G-BA 0 hospitals were not directly recruited, responses from participants working in these hospitals were included in the study because participants satisfied the inclusion criteria of a spatially separate ED. However, maximum EDs in Germany belong to G-BA 1 [34], and the sample in this study had the highest proportion of hospitals from G-BA 3, which are larger hospitals with more infrastructure for communication. Additionally, the highest proportion of participants are from publicly funded hospitals whereas most hospitals in Germany have a commercial sponsor [62]. The division of the sample is also disproportionate for employees with nurses comprising a larger percentage of the sample. Overall, doctors make up only 30.3% of the sample. This disparity may be normal in EDs as the nurse to doctor ratio is high in hospitals [63]. These differences in groups along with broader confidence intervals obtained point towards a lack of precision [64]. However, the results are more robust due to the use of bootstrapping [65].

A major limitation of this study is its cross-sectional nature, due to which only relationships between variables are demonstrated and not causality. A social desirability bias due to self-reported scales may be expected in this study. However, a vital strength is the combination of self- and external assessment of the variable of staff-care. Both versions aid in presenting a clearer representation of the situation [21,59] and can also help in overcoming common method bias [29,30]. Nevertheless, to ensure the confidentiality and anonymity of responses, employee and supervisor data of the same ED were not matched. The VPC scale was originally in English and translated to German, which may have introduced some errors, although the changes were made in agreement with the original author. Additionally, even though the participants were encouraged to answer questions with respect to physical or verbal violence from patients or their attendants, violence was not clearly defined in the questionnaire. This leaves the interpretation of the extent and type of violence to the respondent’s understanding which may be different, making this an important limitation of this study. The questionnaire also does not account for participants who may not have experienced a situation of violence in their department.

This study was also prone to potential non-response bias due to the length of the questionnaire and mandatory nature of all items [66,67], which was used to avoid missing data. This bias may be accentuated due to the recruitment strategy of reaching out to the main contact person of the department or hospital and relying on their time and effort to forward the information to all employees of the ED. A possible bias may have been introduced in the study due to confusion or inattention attributed to reverse-worded items [68]. However, the recoding of these items may have decreased response bias and improved reliability [69].

### 4.5. Implications for Future Research and Practice

#### 4.5.1. Implications for Future Research

Analyzing differences in the perspectives of the VPC and HoL with respect to gender can give more insights into the concepts. Previous research suggests discrepancies in the perception of leadership based on gender [70], and the perception of safety climate [49] as well as on exposure to violence [46]. Further research could also focus on analyzing profession as a control variable in the relationship between HoL and VPC. Continuing research on both these constructs could also incorporate variables like working shift, the type of employment or age, work experience in the ED, and the category of funding received by the hospital, as all these might influence the perception of individuals.

Although the anonymous nature of the survey is an ethical strength, the present study collected data for supervisors and employees from all over Germany without any specifications for teams. In contrast, many previous studies on HoL look at supervisors and employees working in the same team [27,60]. Therefore, future research can be conducted with a focus on specific supervisors and their teams. This would ensure an exhaustive understanding of health-oriented leadership and the violence prevention climate within teams.

#### 4.5.2. Implications for Practice

The results of this study indicate that there is a significant difference in the perspectives of supervisors and employees, as well as doctors and nurses, towards the violence prevention climate. Therefore, there is a need for planning comprehensive violence prevention policies and programs in the EDs with the engagement of all stakeholders, considering and adapting to individual needs. The results further demonstrate that there is a disparity in the staff-care exhibited by the supervisors and the staff-care perceived by the employees. This stresses the need to sensitize supervisors that their employees’ needs for health-oriented leadership might be significantly different from what they perceive and act upon. Therefore, supervisors should be motivated to address health and well-being in their teams in EDs to create a mutual understanding to reduce subjectivity bias [27].

The association of health-oriented leadership with either all or certain dimensions of violence prevention climate emphasizes the critical role of health-oriented leadership training for both supervisors and employees. It can aid supervisors in recognizing employee stress signals and consequently behave in a health-promoting way. On the other hand, employees can learn to be more mindful and explicit about their health needs as well as better recognize the efforts of their supervisors.

## 5. Conclusions

Workplace violence against ED staff by patients and their attendants, in the form of physical violence or verbal aggression, is highly prevalent. Its effects on both the employees and the organization are well described. Although there is a plethora of research that looks at measures to prevent violence, research on the role of leadership is insufficient.

Based on this quantitative analysis, it can be concluded that health-oriented leadership in the form of the self-care and staff-care of both supervisors and employees is associated with different elements of VPC. This study illustrates the association of perceived staff-care with prevention climate, succeeding previous studies that identify leadership as a safety climate antecedent. This study also finds significant differences in how supervisors and employees perceive staff-care and violence prevention climates. A significant difference among doctors and nurses for two dimensions of VPC also supports previous studies where different perceptions of the safety climate are analyzed.

Nonetheless, the results of this study clarify and reinforce the need for the implementation of comprehensive violence prevention programs in EDs that are designed to alleviate the concerns of different population groups working there. Based on the findings of this study, both supervisors and employees can be trained on the concepts of health-oriented leadership to emphasize the importance of caring for their own health (self-care) and, in case of supervisors, for their staff as well (staff-care). This could help enhance their commitment to improving VPC in German EDs, which may lead to a reduction in the exposure to violent events.

## Figures and Tables

**Table 1:** Description of the study population (*n* = 370).

Variables	*n*	%
Gender		
Male	150	40.5
Female	220	59.5
Age		
20–29 Years	84	22.7
30–39 Years	99	26.8
40–49 Years	80	21.6
50–59 Years	92	24.9
60 Years or older	15	4.1
Type of Employment		
Full-time employment (≥35 h/week)	298	80.5
Part-time employment (15–34 h/week)	64	17.3
Part-time or hourly employment (<15 h/week)	8	2.2
Shift *		
Weekdays in day shifts (e.g., early shift or late shift)	297	40.2
Night shifts during the week	166	22.5
Day or night shifts on weekends or holidays	275	37.3
Duration of employment in the current ED **		
<1 year	24	6.7
1–5 years	147	41.3
6–10 years	93	26.1
11–15 years	39	11
>15 years	53	14.9
Total duration of employment in all EDs **		
<1 year	14	3.9
1–5 years	116	32.6
6–10 years	91	25.6
11–15 years	57	16.0
>15 years	78	21.9
Federal Joint Committee [G-BA] level of the hospital		
G-BA level 3	160	43.2
G-BA level 2	103	27.8
G-BA level 1	81	21.9
G-BA level 0	11	3.0
Not known	15	4.1
Category of funding for the hospital		
Private provider (for profit)	63	17.0
Public provider	211	57.0
Independent provider (non-profit)	89	24.1
Not known	7	1.9
Number of beds in the hospital		
Up to 299 beds	76	20.5
300–599 beds	140	37.8
600 or more beds	141	38.1
Not known	13	3.5
State		
Baden-Württemberg	48	13.0
Bavaria	53	14.3
Berlin	20	5.4
Brandenburg	4	1.1
Bremen	14	3.8
Hamburg	9	2.4
Hesse	26	7.0
Mecklenburg-Western Pomerania	6	1.6
Lower Saxony	35	9.5
North Rhine-Westphalia	68	18.4
Rhineland-Palatinate	19	5.1
Saarland	3	0.8
Saxony	8	2.2
Saxony-Anhalt	9	2.4
Schleswig-Holstein	36	9.7
Thuringia	12	3.2

* *n* > 370 because choosing more than one option was possible. ** *n* = 356 as 14 gave implausible answers by responding to have spent more time in their current ED as compared to their whole length of employment.

**Table 2:** Spearman’s correlation coefficients among main study variables for supervisors.

Measure	Mean	SD	1	2	3	4	5
1. Supervisor self-care	3.07	0.61	(0.860)				
2. Supervisor staff-care	3.72	0.54	0.455 ** [0.306, 0.588]	(0.888)			
3. Violence prevention practices and responses	20.87	5.16	0.295 ** [0.133, 0.448]	0.247 ** [0.094, 0.391]	(0.868)		
4. Violence prevention policies and procedures	17.85	5.48	0.232 ** [0.091, 0.377]	0.339 ** [0.199, 0.470]	0.570 ** [0.460, 0.671]	(0.888)	
5. Pressure for unsafe practices	20.40	5.12	0.235 ** [0.067, 0.393]	0.246 ** [0.086, 0.397]	0.480 ** [0.339, 0.611]	0.420 ** [0.282, 0.558]	(0.886)

** *p* < 0.01 level (2-tailed). A 95% bias-corrected and accelerated [BCa] bootstrap confidence interval [CI] reported in brackets, *n* = 164. Cronbach’s alpha in parentheses across the diagonals.

**Table 3:** Spearman’s correlation coefficients among main study variables for employees.

Measure	Mean	SD	1	2	3	4	5
1. Employee self-care	3.18	0.66	(0.850)				
2. Employee assessment of supervisor staff-care	2.32	0.90	0.094 [−0.053, 0.249]	(0.949)			
3. Violence prevention practices and responses	16.81	5.60	0.139 * [0.002, 0.276]	0.289 ** [0.167, 0.415]	(0.875)		
4. Violence prevention policies and procedures	14.76	5.53	0.118 [−0.012, 0.260]	0.287 ** [0.147, 0.429]	0.551 ** [0.443, 0.649]	(0.905)	
5. Pressure for unsafe practices	17.02	4.84	0.250 ** [0.122, 0.372]	0.350 ** [0.219, 0.469]	0.448 ** [0.328, 0.565]	0.398 ** [0.265, 0.515]	(0.877)

** *p* < 0.01 level, * *p* < 0.05 level (2-tailed). The 95% BCa bootstrap CIs reported in brackets, *n* = 204. Cronbach’s alpha in parentheses across the diagonals.

**Table 4:** Parametric group comparisons.

Population	*n*	Mean	SE	*t* (df)	Mean Difference	BCa 95% CI	*p*-Value	Adjusted *p*-Value	Effect Size (*d*)
Violence prevention practices and responses									
Supervisors	166	20.80	0.40	7.05 (368)	3.99	[2.811, 5.145]	0.001	0.025	0.737
Employees	204	16.81	0.39						
Self-care									
Supervisors	166	3.07	0.05	−1.72 (368)	−0.11	[−0.236, 0.008]	0.078	0.050	−0.180
Employees	204	3.18	0.05						
Supervisor Staff-care									
Doctors	75	3.64	0.06	−1.87 (162)	−0.16	[−0.328, 0.021]	0.078	0.025	−0.292
Nurses	89	3.79	0.06						

**Table 5:** Non-Parametric group comparisons.

Population	*n*	Mean Rank	*U*	*z*	99% CI	*p*-Value Monte Carlo Sig. (2-Tailed)	Adjusted *p*-Value	Effect Size (*d*)
Violence prevention practices and responses								
Doctors	112	221.23	10,446.50	−4.24	[0.000, 0.000]	<0.001	0.008	−0.452
Nurses	258	169.99						
Violence prevention policies and procedures								
Doctors	112	188.71	14,089.00	−0.38	[0.703, 0.710]	0.707	0.050	−0.040
Nurses	258	184.11						
Supervisors	166	216.70	11,752.00	−5.07	[0.000, 0.000]	<0.001	0.010	−0.547
Employees	204	160.11						
Pressure for unsafe practices								
Doctors	112	222.44	10,311.00	−4.39	[0.000, 0.000]	<0.001	0.008	−0.469
Nurses	258	169.47						
Supervisors	166	220.09	11,190.50	−5.63	[0.000, 0.000]	<0.001	0.010	−0.612
Employees	204	157.36						
Staff-care								
Supervisors	166	265.41	3458.00	−13.09	[0.000, 0.000]	<0.001	0.010	−1.867
Employees	204	119.45						
Self-care								
Doctors	112	169.10	12,611.50	−1.94	[0.050, 0.054]	0.052	0.017	−0.203
Nurses	258	192.62						
Employee assessment of supervisor staff-care								
Doctors	37	119.66	2454.50	−1.96	[0.048, 0.051]	0.049	0.013	−0.277
Nurses	167	98.70						

**Table 6:** Multiple linear regression models for supervisors.

Variable	Model 1					Model 2					Model 3				
	Violence Prevention Practices and Responses					Violence Prevention Policies and Procedures					Pressure for Unsafe Practices				
	*b*	*SE*	β	*t*	*p*	*b*	*SE*	β	*t*	*p*	*b*	*SE*	β	*t*	*p*
Constant	9.26 (4.60, 13.58)	2.45		3.30	0.001	3.79 (−2.25, 9.06)	2.81		1.28	0.162	10.67 (5.25, 16.43)	2.70		3.78	0.001
Supervisor self-care	1.96 (0.58, 3.30)	0.69	0.23	2.82	0.006	1.40 (0.19, 2.82)	0.72	0.16	1.91	0.048	1.90 (0.30, 3.33)	0.75	0.23	2.72	0.010
Supervisor staff-care	1.51 (0.23, 2.89)	0.66	0.16	1.92	0.024	2.63 (1.05, 4.45)	0.83	0.26	3.17	0.004	1.05 (−0.60, 2.68)	0.83	0.11	1.32	0.211
*R* ^2^	0.111 **					0.126 **					0.086 **				
Adjusted *R*^2^	0.100 **					0.116 **					0.074 **				

(95% BCa CIs reported in parenthesis. Confidence intervals, *p*-values and standard errors based on 1000 bootstrap samples). All values have been rounded off to two decimal places except *p*-values. ** *p* ≤ 0.001. *n* = 164.

**Table 7:** Multiple linear regression models for employees.

Variable	Model 4					Model 5					Model 6				
	Violence Prevention Practices and Responses					Violence Prevention Policies and Procedures					Pressure for Unsafe Practices				
	*b*	*SE*	β	*t*	*p*	*b*	*SE*	β	*t*	*p*	*b*	*SE*	β	*t*	*p*
Constant	9.73 (5.47, 13.81)	2.14		4.84	0.001	10.64 (8.66, 12.71)	1.07		10.33	0.001	7.46 (4.12, 10.89)	1.80		4.49	0.001
Employee self-care	1.09 (−0.036, 2.25)	0.57	0.13	1.89	0.055						1.85 (0.76, 2.88)	0.52	0.25	3.86	0.001
Employee assessment of supervisor staff-care	1.56 (0.64, 2.48)	0.46	0.25	3.68	0.001	1.78 (0.91, 2.62)	0.43	0.29	4.30	0.001	1.59 (0.94, 2.30)	0.33	0.30	4.55	0.001
*R* ^2^	0.087 **					0.084 **					0.168 **				
Adjusted *R*^2^	0.078 **					0.079 **					0.160 **				

(95% BCa CIs reported in parenthesis. Confidence intervals, *p*-values and standard errors based on 1000 bootstrap samples). All values have been rounded off to two decimal places except *p*-values. ** *p* < 0.001. *n* = 204.

**Table 8:** Summary of regression models.

Model	Independent Variables	Dependent Variables	Cohen *f*^2^	Adjusted *p*-Value	Power
Model 1	Supervisor self-care Supervisor staff-care	Violence prevention practices and responses	0.111	0.025	0.949
Model 2		Violence prevention policies and procedures	0.131	0.017	0.969
Model 3		Pressure for unsafe practices	0.080	0.050	0.905
Model 4	Employee self-care Employee assessment of supervisor staff-care	Violence prevention practices and responses	0.085	0.050	0.967
Model 5		Violence prevention policies and procedures	0.092	0.025	0.980
Model 6		Pressure for unsafe practices	0.190	0.017	>0.999

## Data Availability

The datasets generated and analyzed during the current study are not publicly available due to German national data protection regulations but are available from the corresponding author on reasonable request.

## Supplementary Materials

The following supporting information can be downloaded at: https://www.mdpi.com/article/10.3390/healthcare11162234/s1, Table S1: Stratified sampling technique; Table S2: Reliability analysis of health-oriented leadership instrument; Table S3: Assumption testing for multiple linear regression; Table S4: Descriptive statistics per sub-scale of HoL; Table S5: Descriptive statistics of main six variables; Table S6: Descriptive statistics for groups per position in ED for VPC variables; Table S7: Descriptive statistics per professional group for VPC variables; Table S8: Descriptive values per group for hypothesis testing for HoL variables; Table S9: Holm–Bonferroni corrections for doctors and nurses; Table S10: Holm–Bonferroni corrections for supervisors and employees; Table S11: Holm–Bonferroni corrections for supervisors (regression analysis); Table S12: Holm–Bonferroni corrections for employees (regression analysis); Table S13: Cross-validation of regression models.
