# Supplementary material for: Violence Prevention Climate and Health-Oriented Leadership in German Emergency Departments
Source: Healthcare (Basel). 2023 Aug 8;11(16):2234. doi: 10.3390/healthcare11162234 (PMC10454408; doi:10.3390/healthcare11162234)
Supplement: Supplementary file 1 [file healthcare-11-02234-s001.zip › healthcare-2479088-supplementary.pdf]

Table S1. *Stratified sampling technique*

G-BA level	Number of EDs per G-BA level (*a*)	Proportion of each category in the total (*b* = *a*/1065*100)	Number of EDs in each category to be included (*c* = *b**200)
1	633	59%	118
2	265	25%	50
3	167	16%	32

Table S2. *Reliability analysis of Health-oriented Leadership instrument*

	Awareness	Value	Personal lifestyle	Work behaviour	Complete scale
Self-care	.826 (6)	.747 (3)	.866*(2)	.719 (8)	.853 (19)
Supervisor staff-care	.797 (6)	.829 (3)	.846 (3)	.871 (10)	.908 (22)
Employee assessment of supervisor staff-care	.880 (6)	.898 (3)	.869 (3)	.914 (10)	.949 (22)

Number of items in each subscale in parenthesis.
*Spearman Brown coefficient value.

Table S3. *Assumption testing for multiple linear regression*

Models	Linearity	Indepen-dence of residuals	Homosc-edasticity	Normally distributed errors	Multico-llinearity	Outliers	Influential cases
Model 1	Yes	2.163	Yes	Approximately normal	1.231	9	.055
Model 2	Yes	1.795	Yes	Approximately normal	1.231	8	.047
Model 3	Yes	2.171	Yes	Nearly approximately normal	1.231	5	.093
Model 4	Yes	1.878	Yes	Approximately normal	1.016	5	.082
Model 5	Yes	1.903	Yes	Approximately normal	1.016	6	.067
Model 6	Yes	1.655	Yes	Approximately normal	1.016	12	.101

Table S4. *Descriptive statistics per sub-scale of HoL*

	M	SD	Reported minimum	Reported maximum
**Supervisor Self-care**				
Awareness	3.40	0.84	1.50	5.00
Value	2.91	0.93	1.00	5.00
Personal lifestyle	3.15	1.17	1.00	5.00
Work Behaviour	1.85	0.66	1.38	4.75
**Employee Self-care**				
Awareness	3.45	0.94	1.17	5.00
Value	3.32	1.04	1.00	5.00
Personal lifestyle	3.40	1.23	1.00	5.00
Work Behaviour	2.87	0.70	1.00	4.63
**Supervisor Staff-care**				
Awareness	3.82	0.63	1.00	5.00
Value	4.54	0.65	1.67	5.00
Personal lifestyle	3.10	1.14	1.00	5.00
Work Behaviour	3.54	0.73	1.00	5.00
**Employee Staff-care**				
Awareness	2.49	1.07	1.00	5.00
Value	2.87	1.28	1.00	5.00
Personal lifestyle	1.72	0.99	1.00	5.00
Work Behaviour	2.23	0.97	1.00	5.00

Table S5. *Descriptive statistics of main six variables*

		Self-care	Staff-care by supervisors	Employee assessment of supervisor staff-care	Practices and responses	Policies and procedures	Pressure for unsafe practices
N	Valid	370	164	204	370	370	370
	Missing	0	206	166	0	0	0
Mean		3.13	3.72	2.32	18.60	16.14	18.49
Median		3.05	3.73	2.23	19.00	16.00	18.00
Std. Deviation		0.64	0.54	0.90	5.76	5.70	5.25
Minimum		1.63	2.27	1.00	6	6	6
Maximum		4.89	4.86	4.82	30	30	30

Missing values because of specificity of target population.

Table S6. *Descriptive statistics for groups per position in ED for VPC variables*

Variables		*M*	*Mdn*	*SD*	Skewness	Kurtosis
Violence prevention practices and responses	Supervisor	20.80	21.00	5.19	-0.316	-0.239
	Employee	16.81	17.00	5.59	-0.092	-0.610
Violence prevention policies and procedures	Supervisor	17.83	18.00	5.47	-0.109	-0.369
	Employee	14.76	15.00	5.53	0.172	-0.640
Pressure for unsafe practices	Supervisor	20.30	19.00	5.19	0.163	-0.621
	Employee	17.02	17.00	4.84	0.100	-0.023

Supervisors, *n* = 166, Employees, *n* = 204.

Table S7. *Descriptive statistics per professional group for VPC variables*

Variables		*M*	*Mdn*	*SD*	Skewness	Kurtosis
Violence prevention practices and responses	Doctor	20.44	21.00	5.61	-0.591	0.065
	Nurse	17.81	18.00	5.65	-0.074	-0.491
Violence prevention policies and procedures	Doctor	16.31	16.00	5.74	0.037	-0.510
	Nurse	16.07	16.00	5.70	0.038	-0.631
Pressure for unsafe practices	Doctor	20.14	19.00	4.90	-0.099	-0.047
	Nurse	17.78	17.00	5.251	0.334	-0.065

Doctors, *n* = 112, Nurses, *n* = 258.

Table S8. *Descriptive values per group for hypothesis testing for HoL variables*

Variables		*n*	*M*	*Mdn*	*SD*	Skewness	Kurtosis
Staff-care	Supervisor	164	3.72	3.73	0.54	-0.383	0.030
	Employee	204	2.32	2.23	0.90	0.626	-0.203
Self-care	Supervisor	166	3.07	3.05	0.61	0.296	-0.247
	Employee	204	3.18	3.05	0.66	0.265	-0.291
Self-care	Doctor	122	3.02	3.00	0.54	0.250	-0.138
	Nurse	258	3.17	3.11	0.67	0.235	-0.399
Staff-care by supervisors	Doctor	75	3.64	3.68	0.55	-0.571	-0.002
	Nurse	89	3.79	3.82	0.52	-0.189	-0.141
	Doctor	37	2.53	2.59	0.77	0.397	-0.272

Employee on supervisor staff-care	Nurse	167	2.27	2.18	0.92	0.712	-0.115

Table S9. *Holm-Bonferroni corrections for doctors and nurses*

Population groups	Hypothesis	*p*-value	Rank	Holm-Bonferroni value
Doctors and nurses	Practices	0.00001	1	0.008
	Policies	0.707	6	0.050
	Pressure	0.00001	1	0.008
	Self-care	0.052	4	0.017
	Supervisor staff-care	0.078	5	0.025
	Employee assessment of supervisor staff-care	0.049	3	0.013

Table S10. *Holm-Bonferroni corrections for supervisors and employees*

Population groups	Hypothesis	*p*-value	Rank	Holm-Bonferroni value
Supervisors and employees	Practices	0.001	4	0.025
	Policies	0.00E+00	1	0.010
	Pressure	0.00E+00	1	0.010
	Staff-care	0.00E+00	1	0.010
	Self-care	0.078	5	0.050

Table S11. *Holm-Bonferroni corrections for supervisors (regression analysis)*

Models	*p*-value	Rank	Holm-Bonferroni value
Model 1	0.00008	2	0.025
Model 2	0.000019	1	0.017
Model 3	0.000744	3	0.050

Table S12. *Holm-Bonferroni corrections for employees (regression analysis)*

Models	*p*-value	Rank	Holm-Bonferroni value
Model 4	0.000103	3	0.050
Model 5	0.000027	2	0.025
Model 6	8.95E-09	1	0.017

Table S13. *Cross-validation of regression models*

Models	Difference between R^2 and adjusted R^2 from SPSS	New adjusted R^2 using Stein's formula	Difference between R^2 and new adjusted R^2
Model 1	1.1%	.083	2.8%
Model 2	1.0%	.099	2.7%
Model 3	1.2%	.057	2.9%
Model 4	0.9%	.064	2.3%
Model 5	0.5%	.070	1.4%
Model 6	0.8%	.147	2.1%
